# Efficacy and Feasibility of Proton Beam Therapy in Relapsed High-Risk Neuroblastoma-Experiences from the Prospective KiProReg Registry

**DOI:** 10.3390/curroncol29110649

**Published:** 2022-10-30

**Authors:** Danny Jazmati, Barbara Hero, Theresa M. Thole.-Kliesch, Julien Merta, Hedwig E. Deubzer, Christian Bäumer, Feline Heinzelmann, Stefanie Schulze Schleithoff, Friederike Koerber, Angelika Eggert, Rudolf Schwarz, Thorsten Simon, Beate Timmermann

**Affiliations:** 1 Department of Particle Therapy, University Hospital Essen, West German Proton Therapy Centre Essen (WPE), West German Cancer Center (WTZ), 45147 Essen, Germany; 2 Children’s Hospital, University of Cologne, 50937 Cologne, Germany; 3 Department of Pediatric Oncology/Hematology, Charité-Universitätsmedizin, 10117 Berlin, Germany; 4 West German Proton Therapy Centre Essen (WPE), West German Cancer Center (WTZ), 45147 Essen, Germany; 5 Department of Physics, TU Dortmund University, 44227 Dortmund, Germany; 6 Department of Radiology, University of Cologne, 50937 Cologne, Germany; 7 Department of RT and Radiooncology, Outpatient Center, University Medical Center Hamburg-Eppendorf, Martinistraße 52, 20246, 20251 Hamburg, Germany

**Keywords:** Neuroblastoma, childhood cancer, proton beam therapy

## Abstract

Background: Despite an intensive multimodal treatment approach, approximately 50% of high-risk (HR) neuroblastoma (NB) patients experience progression. Despite the advances in targeted therapy, high-dose chemotherapy, and other systemic treatment options, radiation therapy (RT) to sites of relapsed disease can be an option to reduce tumor burden and improve chance for disease control. Methods: Patients who received salvage irradiation with proton beam therapy (PBT) for local or metastatic relapse of HR NB within the prospective registry trials KiProReg and ProReg were eligible for this retrospective analysis. Data on patient characteristics, multimodality therapy, adverse events, and oncologic endpoints were evaluated. Adverse events were assessed before, during, and after PBT according to common terminology criteria for adverse events (CTCAE) V4.0. Results: Between September 2013 and September 2020, twenty (11 male; 9 female) consecutive patients experiencing local (*N* = 9) or distant recurrence (*N* = 25) were identified for this analysis. Distant recurrences included osteomedullary (*N* = 11) or CNS lesions (*N* = 14). Salvage therapy consisted of re-induction chemo- or chemo-immuno-therapy (*N* = 19), surgery (*N* = 6), high-dose chemotherapy and stem cell transplantation (*N* = 13), radiation (*N* = 20), and concurrent systemic therapy. Systemic therapy concurrent to RT was given to six patients and included temozolomide (*N* = 4), carboplatine (*N* = 1), or anaplastic lymphoma kinase tyrosine kinase inhibitors (ALK-TKI) (*N* = 1). A median dose of 36 Gy was applied to the 34 recurrent sites. Local RT was applied to 15 patients, while five patients, received craniospinal irradiation for CNS relapse. After a median follow-up (FU) of 20 months (4–66), the estimated rate for local control, distant metastatic free survival, and overall survival at 3 years was 68.0%, 37.9%, and 61.6%, respectively. During RT, ten patients (50%) presented with a higher-grade acute hematologic adverse event. Late higher-grade sequelae included transient myelitis with transverse section (*N* = 2) and secondary malignancy outside of the RT field (*N* = 1). Conclusion: Our study demonstrates the efficacy and safety of RT/PBT for recurrent HR NB in a multimodality second-line approach. To better define the role of RT for these patients, prospective studies would be desirable.

## 1. Introduction

Neuroblastoma (NB) is the most common extracranial malignancy among children [1]. Patients are treated in different risk groups based on age, stage, and molecular pathology. Patients diagnosed at > 18 months or > 12 months of age (depending on the study protocol) with evidence of either MYCN amplification or distant metastases are defined as high-risk. Of all children with NB, approximately 30–50% are defined as high-risk (HR) disease. Standard of care for these patients is induction chemotherapy, surgery, high-dose chemotherapy with stem cell transplantation, radiotherapy (RT) to the preoperative tumor bed, and immunotherapy with dinutuximab beta. Despite the tremendous advances achieved due to this intensive multimodality treatment approach, unfortunately progression is experienced in more than half of those children, mainly within two years after diagnosis [2,3].

In general, the prognosis for patients with recurrent HR NB is poor, with survival rates reported between 3% and 15% only [4,5]. However, with consequent second-line induction chemotherapy, combined immune-chemotherapy, parallel targeted therapies, and consolidation with haploidentical stem cell transplantation, there are promising opportunities available today [6,7,8]. Nevertheless, there are no large interdisciplinary trials except the RIST rNB2011 trial that has currently finished patient enrolment. Therefore, decision making on relapse treatment is particularly challenging and highly individualized. While RT is an integral part of the first-line treatment, the role of RT in relapsed HR NB is still controversial as not being supported by convincing evidence. 

Virtually all HR NB recurrences are resistant to elements of standard systemic therapy. Individualized multimodal treatment concepts integrating RT early, especially in local or oligometastatic relapse, could contribute to the induction of an overall response to therapy. However, considering the combined treatment burden of first- and second-line treatment, RT represents a major challenge in children with relapsed NB. Due to its unique physical properties, PBT offers an opportunity to reduce the toxicity of RT [9,10].

Here, we report our retrospective institutional experience on the efficacy and safety of local ablative PBT within the multimodal treatment approach for children with relapsed HR NB.

## 2. Materials and Methods

### 2.1. Patients

Patients irradiated for local or distant relapsed HR NB were included in this study. All patients selected for analysis were enrolled in two prospective registries (DKRS00005363 and DRKS00004384) collecting data on patients, treatment, survival, and toxicity. Both adults and children were eligible for this analysis. Consistent with the Helsinki Declaration and its subsequent amendments, consent to participate in the respective registries was obtained from all legal guardians. Both prospective registry studies were approved by the local ethics committee.

### 2.2. Treatment

As described in more detail already elsewhere, initial management included induction chemotherapy, surgery, high-dose chemotherapy for consolidation, and post-consolidation treatment either with retinoic acid or dinutuximab [11]. Prior to 2018, radiation was reserved for residual MIBG-positive tumors after surgery and high-dose chemotherapy, according to the national “NB 2004 Trial Protocol for Risk Adapted Treatment of Children with Neuroblastoma” (NB 2004/NB2004-HR) (NCT 00410631; NCT 00526318). Since 2018, the preoperative tumor extension was irradiated up to 21.6 Gy in all HR NB patients with an additional boost to residual disease to a cumulative dose of 36 Gy. In this cohort, radiotherapy was already given as part of the first-line treatment in five patients. 

**Figure 1:**
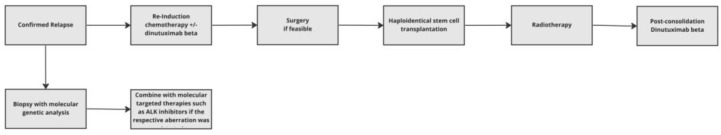
Flow diagram of our interdisciplinary standard of care for relapsed neuroblastoma.

The standard recurrence strategy was composed of re-induction therapy, surgery if considered feasible without causing relevant morbidity, high-dose chemotherapy followed by stem cell transplantation, irradiation, and administration of dinutuximab beta (Figure 1).

For all patients, overall treatment strategies were determined by a multidisciplinary tumor board (MDT) within the framework of the national German NB Board composed of pediatric oncologists, pediatric surgeons, and representatives of proton and photon RT. All relapses were confirmed and assessed during by the national reference radiology. In general, at the time of progression, a biopsy with molecular genetic analysis was recommended. Usually, treatment consisted of re-induction therapy, followed by haploidentical stem cell transplantation, RT, and post-consolidation treatment with the monoclonal antibody dinutuximab beta. Re-induction therapy consisted of either systemic chemotherapy or a combination of chemotherapy and dinutuximab beta. The second-line treatment was combined with molecular targeted therapies such as ALK inhibitors if the respective aberration was detected [8]. The possibility of resection was assessed individually for each case. The decision for PBT was determined by the German NB Board. PBT was generally preferred for patients of young age and tumor tissue at critical localization. A side-separated renal scintigraphy was performed before retroperitoneal RT potentially involving the kidneys. The interdisciplinary team for planning and treating consisted of physicists, radiation oncologist, social workers, pediatric anesthesiologists, psychologists, and pediatric oncologist. The interdisciplinary team involving the patient and legal guardians individually evaluated the need for sedation. If anesthesia was required for immobilization and treatment, a deep sedation was carried out by a pediatric anesthesiologist. 

Radiotherapy for relapsed disease was performed similar to the first-line approach. According to our local standards, each patient was scheduled for planning CT using a slice thickness of 1 mm and a planning MRI. Imaging was fused with diagnostic images. The standard approach was to irradiate the preoperative tumor extent or, if no surgery was performed, the extent after induction chemotherapy with 21.6 Gy followed by a boost to the residual tumor at the time of radiotherapy up to 36 Gy. In non-central nervous system (CNS) progression, tumor extent at the time of recurrence was adjusted for current anatomy and extended for microscopic tumor extension using a CTV margin of 0.5 cm. Residual sites at the time of RT were defined as boost volume. In case of CNS recurrence, the complete subarachnoid space was defined as the CTV and extended by a 3–5 mm margin to create the PTV. Subsequently, the CNS lesion or, in the case of surgery, the resection cavity, was defined as the boost volume. Standard irradiation was 21.6 Gy followed by a boost to 36 Gy on residual tumor tissue. The tumor bed of CNS lesions was boosted up to 36 Gy regardless of the presence of residual tumor lesions. A generic relative biological effectiveness (RBE) factor of 1.1 (relative to that of Co-60) was assumed for all dose concepts. Proton doses were expressed in terms of Gy (RBE) (Gy (RBE) = proton Gy X 1.1). Treatment doses were calculated using RayStation © Version 7.0 (RaySearch Laboratories, Stockholm, Sweden). Each patient underwent a laboratory examination including liver and kidney parameters as well as a differential blood count at the start of therapy and weekly during RT. The haematological toxicity was classified according to common terminology criteria for adverse events (CTCAE) version four.

### 2.3. Follow-Up

Acute and late adverse events were classified according to CTCAE Version 4.0. Complications within 3 months after starting PBT were considered acute toxicity. Thereafter, complications were defined as late complications. Within the prospective registry, adverse events were assessed before, during RT (weekly), after 90 days, and then at least annually. Adverse events above CTCAE 2 were defined as higher grade complications. Patients underwent clinical examination, tumor marker assessment, bone marrow examination, and cross-sectional and functional diagnosis including MRI and MIBG during follow up (FU). 

### 2.4. Plan Analysis

The cases with unexpected side effects were retrospectively recalculated with respect to the variable relative biological effectiveness (RBE) weighted dose distribution. These computations could reveal correlations between high-LET dose depositions and increased toxicity. For this purpose, an in-house script was used for the Monte Carlo-based calculation, which is implemented within the research version RayStation 9A IonPG (v. 8.99.30.101). α\/β = 10 Gy for the target volume, and α\/β = 2 “Gy” for the delineated myelon and for the normal tissue, which corresponds to the body ROI minus the target, are set for the tissue radiosensitivity values in RayStation. Concerning a conservative tissue sensitivity for the margin around the CTV, the RBE calculation with the tissue-specific radiosensitivity was applied for the whole PTV. The RBE model used is the Wedenberg model [12]. The final calculation was performed with a statistical uncertainty of the Monte Carlo simulation of 0.5%.

### 2.5. Statistical Analysis

Qualitative data was presented as frequency (minimum–maximum) and percentage. Cut-off was based on the known cut-off or median. Local recurrence was used to describe failure rates at irradiated sites. Accordingly, local control was defined as the absence of local recurrence. Distant failure was defined as metastatic recurrence at a non-irradiated site. Local recurrence-free survival (LRFS), metastasis-free survival (MFS), and overall survival (OS) were calculated from the time of relapse and plotted according to the Kaplan–Meier method. Patients were censored at the time of last follow-up if they had no event. All statistical analyses were performed using R (v. 4.1.0, 18 May 2021, R Core Team, 2021).

## 3. Results

### 3.1. Patients and Tumor Characteristics

A total of 20 patients (11 male; 9 female) were included in the analysis. All subjects initially presented with HR NB at diagnosis and subsequently experienced progression. High-risk was defined by N-myc amplification or disseminated disease in patients over 18 months of age. At the time of relapse, patients had a median age of 6.3 years (range, 1.05–19.08). The median time between initial diagnosis and recurrence was 35.5 months (range, 9–189). Table 1 summarizes information on patients and treatment characteristics.

### 3.2. Initial Treatment

Patients had a median age of 2.98 years (range, 0.67–15.16) at the time of first diagnosis. All but one patient underwent re-induction therapy. Re-induction therapy was applied in the majority of cases according to the RIST rNB2011 trial protocol (NCT01467986) (*N* = 12) or with a combination of irinotecan, temozolomid, and dinutuximab beta without GM-CSF (*N* = 3) [13]. Surgery was performed in six cases. This was usually followed by high-dose chemotherapy (*N* = 13) with subsequent autologous (*N* = 4) or haploidentical stem cell transplantation (*N* = 9). Although stem cell transplantation constituted the recommended approach, seven patients did not receive a stem cell transplantation based on the individual decision of the respective centre.

Radiotherapy was administrated to all relapse lesions that were present after re-induction chemotherapy. In median, one target volume (range, 1–5) was irradiated. Table 2 displays the details. Patients were irradiated with a mean total dose of 36Gy (range, 21.6 Gy–39.6 Gy) (RBE). In one case receiving craniospinal irradiation (CSI), the initial preoperative tumor bed was included in the radiation field as the first-line therapy, which was less than two years ago and did not include RT.

In five cases, the first and second irradiation volume overlapped. In two cases, re-irradiation was performed due to local progression 22 and 55 months after initial irradiation. In one of the patients, despite a second irradiation, another local progression occurred within the radiation field 33 months after diagnosing the recurrence. In three other patients, CSI was initiated 6, 12, and 19 months after radiotherapy of the primary site during first-line therapy. In all five cases, radiation burden to organs at risk from first and second irradiation occurred. The median D1 dose to the spinal cord in all patients was 45.45 Gy (RBE) (range, 41.6 Gy–49.6 Gy). The median dose to both kidneys was kept below 10 Gy in all cases. In one case with intracranial re-irradiation after and interval of 22 months, a cumulative D1 dose of 77.8 Gy (RBE) to the right optic nerve was accepted while sparing the contralateral optic nerve (cumulative D1: 35.2 Gy RBE) and the chiasm (cumulative D1 33.0 Gy RBE). Concurrent with RT, six patients received additional systemic therapy, such as temozolomide (*N* = 4), carboplatine (*N* = 1), or the anaplastic lymphoma kinase (ALK)–Tyrosine Kinase Inhibitors (TKI) lorlatinib (*N* = 1). 

### 3.3. Outcome

**Figure 2:**
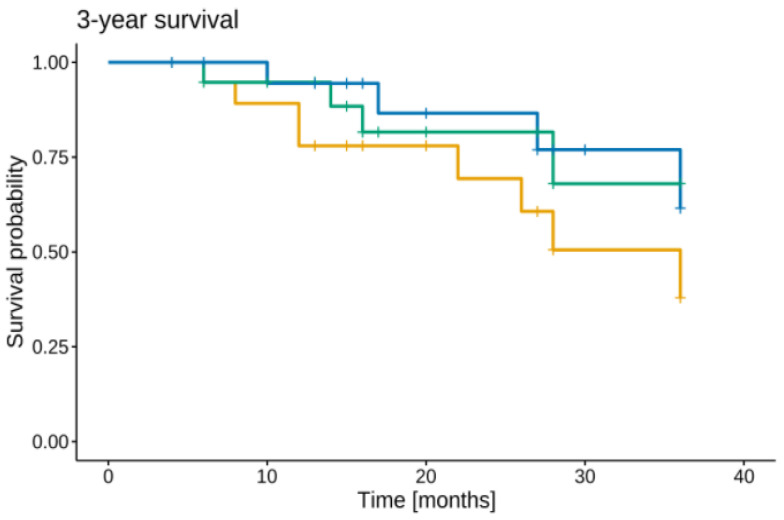
Kaplan–Meier estimates of local control (LC), Distant metastatic free survival (DMFS), and survival (OAS), respectively, for all patients. DMFS = 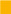, LC = 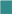, OS = 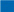.

**Figure 3:**
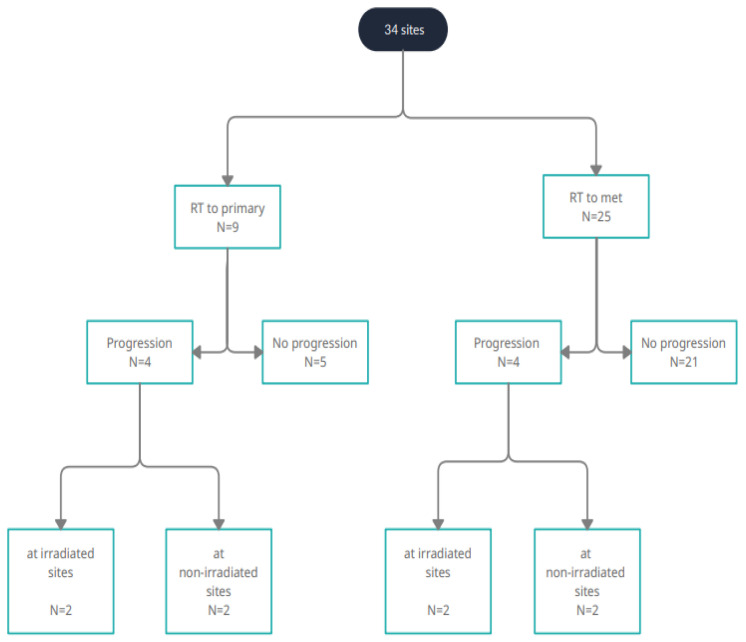
Flow diagram for recurrent sites of neuroblastoma patients. Radiotherapy (RT) was given to primary and metastatic (met) recurrent sites.

After a median follow-up (FU) time of 20 months (range, 4–66 months), the estimated three-year LRFS, MFS, and OS rates from relapse were 68.0%, 37.9%, and 61.6%, respectively (Figure 2). Of eight patients who presented with further progression after relapse therapy, four patients experienced combined local and systemic recurrence, whereas four children experienced disseminated progression. The pattern of recurrence is illustrated in Figure 3. None of the treated children experienced local recurrence only. Out of four patients receiving CSI for CNS progression, one patient developed progression outside of the CNS. So far, three patients remained in remission at last FU of 6, 20, and 36 months after initiation of relapse treatment, respectively (Table 2).

### 3.4. Adverse Events

No higher-grade acute toxicity attributable to RT was observed other than hematologic toxicity. At baseline, patients revealed a mean WBC, HB, and platelet count of 4500 /mm³, 11.1 g/dL, and 183,000 /mm³, respectively. On average, blood counts decreased to a nadir of 1780 /mm³, 9.9 g/dL, and 125,000 /mm³, respectively. Among the entire cohort, ten patients experienced higher-grade hematologic adverse events including two patients who received CSI and three who were treated with temozolomide concomitant to RT. One patient experienced a central line infection during treatment. 

Three patients developed unexpected late events of unknown cause. Two of them experienced myelopathy with paraplegia (CTCAE grade 3, CTCAE grade 4). Another patient presented with a secondary cancer after two abdominal irradiations outside of the radiation field when developing a sarcoma at the scapula.

Among the two patients with myelopathy, one patient had received dinutuximab four weeks after RT and developed marked inflammatory reaction with capillary leak syndrome during dinutuximab beta infusion and neuropathy seven months after the dinutuximab therapy. This patient developed paraplegic symptoms with neurogenic bladder dysfunction and paraplegia involving both legs and corresponding MRI changes with evidence of myelopathy within the RT field. However, the clinical situation of this patient improved substantially during follow-up. The second patient received dinutuximab six weeks after RT. This patient presented 13 months after RT with urinary and stool incontinence and paraplegia of the lower limbs. MRI revealed homogeneous enhancement in the cauda equine. High-dose corticosteroids did not improve the condition. While in the first case busulfan plus melphalan was not a component of the overall multimodal relapse treatment concept, in the second, busulfan plus melphalan was applied during first-line treatment 13 months before radiotherapy during second-line treatment and 25 months prior the onset of symptoms (Table 3). In both cases, neither D 0.1% nor D 1% was found to exceed a dose limit of 50 Gy even when a variable RBE was considered (Table 4)

## 4. Discussion

This study investigates the role of local and metastatic salvage irradiation within a multimodality treatment approach for recurrent HR NB. With acceptable toxicity, multi-modality treatment achieved encouraging tumor control rates with a three-year LC and OS of 68% and 61.6%, respectively. Although in principle, NB is a radiosensitive malignancy, data on RT for relapsed patients are scarce. Our data on external beam irradiation for local and metastatic relapse including CNS in twenty relapsed HR NB patients are therefore unique and important to further optimizing salvage strategies.

So far, only a few studies investigated irradiation for local or metastatic recurrence. Dove et al. reported on a cohort of 20 HR NB patients treated for loco-regional relapse [14]. Ten of these twenty patients received RT with a median total dose of 27 Gy. The authors revealed that RT significantly (*p* = 0.04) reduced the risk of subsequent local-regional failure. Rich et al. investigated 44 patients with recurrent or refractory NB treated with intraoperative RT (IORT) after GTR [15]. After a median FU of 10.5 months, LC and OS rates were 50.4% and 23.4%, respectively. However, the authors reported a considerable complication rate, raising the question of whether IORT is the optimal treatment modality for these heavily pre-treated patients. Of note is that intraoperative radiotherapy never became a standard element of neuroblastoma treatment. In contrast, our study achieved both a more favourable tumor outcome and better feasibility compared to the two aforementioned studies. In contrast to these other studies, our study included metastatic recurrences as well. Our median follow-up of 20 months was comparable to that of Rich et al. having a median follow-up of 10 months. However, our median follow up was considerably shorter than the median follow-up of 13 years reported in Dove et al. Consequently, varying lengths of follow-up hamper comparability of the studies and allow only limited conclusions to be drawn. 

However, use of local radiotherapy has the potential to reduce the burden of treatment-resistant cells after initiation of systemic therapy. Conceivably, radiotherapy may lead to anti-tumor immunity through the release of tumor antigens, the proliferation of T-lymphocytes. 

The treatment of CNS recurrences poses a particular challenge. A recent analysis of the relapses after first-line therapy in European SIOP-Europe (SIOPEN) High-Risk Neuroblastoma Study 1 (HR NB 1) (NCT01704716) described a median OS after CNS recurrence of only four months. Less than 10% of the patients survived longer than 3 years [16]. Moreover, Matthay et al. reported that all children with CNS recurrence died after a median survival time of two months. In a study from St. Jude Children’s Hospital, two of four patients treated with CSI were alive 50 and 62 months after diagnosis without experiencing any recurrence [17,18]. Croog et al. retrospectively compared patients treated with CSI and intraventricular immune radiotherapy to those who only had local radiotherapy. A total of 12 patients (75%) in the CSI group were alive after a median follow-up of 28 months, while all 13 patients in the non-CSI group died after a median of 8.8 months [19]. Recently, Luo et al. reported on 94 patients with CNS recurrence who received CSI with a total dose of 18 or 21 Gy in a multimodal therapy concept including surgical resection, chemotherapy with temozolomide and irinotecan, and intraventricular compartmental radioimmunotherapy in addition to CSI. The authors reported a five-year OS rate in the 18 Gy or 21 Gy group of 43% and 47%, respectively. No significant impact of the total CSI dose on outcome was observed. In our study, three out of four patients achieved disease when receiving CSI for CNS recurrence. Although our cohort is too small to be conclusive, our data seem to support the previous literature suggesting that CSI has a role for patients with CNS recurrence. Taking into account the limited prognosis, evaluating the feasibility of radiation therapy is of great importance, both on short and on long term.

Considering the multimodality treatment burden, close monitoring of the patient’s bone marrow function remains important. Patients can be at risk for significant hematotoxicity affecting not only quality of life, but also jeopardizing an uninterrupted treatment course. Additionally, radiation fields that include large parts of the bone marrow very likely increase hematologic toxicity. Therefore, it is not surprising that a drop in blood counts in a large proportion of patients occurred. Furthermore, the hematologic reserve has a major role when considering simultaneous systemic therapy. The efficacy of temozolomide, in combination or alone, has been established for NB [20,21]. In three patients, we applied chemotherapy with temozolomide according to the regimen published by Stupp et al. for glioblastoma [11]. We observed myelodepression including CTCAE grade 4 thrombocytopenia in all patients [11]. Therefore, parallel administration with temozolomide during RT appears to be challenging. In our study, other concurrent therapies included carboplatin and the ALK inhibitor lorlatinib. Since ALK mutations were detected on NB cells in both primary and relapse, an attempt was made to extrapolate the achievements gained from ALK inhibitors in non-small lung cancer therapy to NB [22]. Two retrospective studies in adult patients with non-small lung cancer showed no higher-grade complications attributable to the combined administration of ALK inhibitors and RT [23,24]. However, given the common presence of hepatic impairment in children with NB, close examination, and monitoring of liver function seems essential prior to initiation of combined therapy. ALK Tyrosine Kinase Inhibitor Therapy was combined with RT only in one patient in the present study. However, no increased toxicity was observed. Unfortunately, this patient experienced early combined local and systemic progression 6 months after initiation of second-line treatment. 

Regarding late effects, we observed two patients presenting with myelopathies matching with the irradiation field. Our group had previously already published one case with manifestations of a paraplegic syndrome correlating with the radiation field during adjuvant dinutuximab therapy within first-line therapy [25]. Another comparable case was published by Ding et al. [25,26]. This current study provides further evidence of a possible adverse effect of combining radiation and dinutuximab in the treatment of NB potentially resulting in an increasedIn Proceedings of the risk of myelopathy. In the light of this suspect, treatment planning needs to aim for a particularly low dose exposure to the myelon, obviously even below the known tolerance doses if adjuvant treatment with dinutuximab is planned. Although we had already established an interval between irradiation and radiotherapy of at least four weeks in cases with a radiation volume interfering with the CNS, obviously these adverse effects can occur despite taking these precautions. As these reported events are not fully understood yet, any combination of RT with new drugs needs to be used with caution and optimally in the frame of a clinical trial.

An additional challenge in second-line treatment is re-irradiation. Only a minority of this cohort had radiotherapy as part of their first-line treatment. Re-irradiation was provided to only two of the included patients. In three others, first and second irradiation field overlapped. While the previous GPOH trial recommended radiotherapy as part of first-line therapy only for MIBG-positive residual tumors, current concepts include irradiation of the preoperative tumor extent regardless of the presence of residual tumor. Thus, in the future, it will be even more important to better understand the role of re-irradiation for NB regarding efficacy and safety. Today, cumulative dose limits for organs are not defined in childhood and represent a major challenge. In our cohort, none of the cases experienced higher grade complications. 

Our study has several limitations. First, we have to acknowledge that this study was retrospective. In addition, the number of patients was small, limiting the statistical power. Furthermore, only patients receiving RT were analyzed not allowing any comparison with patients without RT potentially introducing a selection bias. 

Despite those limitations, we are convinced that the study adds to the important field of strategies in recurrent NB.

## 5. Conclusions

Overall, our study demonstrated good feasibility and results can promote RT for local therapy in relapsed HR NB patients. In the future, it would be desirable to obtain prospective data to better define the role of RT and the risk for late effects for this critical cohort. In this rapidly evolving field, the feasibility of radiation therapy combined with new drugs will continue to be of concern and matter of clinical studies.

## Figures and Tables

**Table 1:** Patient and treatment characteristics.

Characteristics		%
**sex**	***n***	
male	12	60
female	8	40
N-myc amplification		
yes	15	
no	5	
**INSS Stage 4 at first diagnosis**		
Yes	18	
No	2	
**age at relapse**	**months**	
median	73.5	
range	26.0–221.0	
**Re-Induction**	***n***	
RIST	13	65
Immunochemotherapy with irinotecan/temozolomide day 1–5 and dinutuximab beta day 2–6	2	10
other	4	20
none	1	5
**Resection of relapse**	***n***	
yes	14	70
no	6	30
**Bone Marrow Transplant at relapse**	***n***	
none	7	35
autologous stem cell transplant	4	20
haploidentical stem cell transplant	9	45
**age at start of proton therapy**	**months**	
median	85	
range	22–224	
**Median number of fractions**	20	
**Consolidation therapy**	***n***	
Immunotherapy	10	50
temozolomide	4	20
none	6	30

N: number, %: percent, RIST: Multimodal Molecular Targeted Therapy to Treat Relapsed or Refractory High-risk Neuroblastoma (NCT01467986), PBT: Proton beam therapy Gy: Gray.

**Table 2:** Overview of the applied treatment.

Case	Site	Number and Location	Dose, Target	Relapse I Side	Relapse U.I. Side	Death 1 = Yes 0 = No
Case 1	P	1	21.6 Gy; L	0	0	0
	M	0	-			
Case 2	P	1	39.6 Gy; L	0	0	0
	M	0	-			
Case 3	P	0	-		0	0
	M	1 (CNS)	36 Gy; CSI	0		
Case 4	P	0	-		1	1
	M	1 (bone)	36 Gy; L	1		
Case 5	P	0	-		0	0
	M	2 (bone)	36 Gy; L	0		
Case 6	P	1	36 Gy, L	0	1	1
	M	0	-			
Case 7	P	0	-	-	0	0
	M	5 (CNS)	36 Gy; CSI	0		
Case 8	P	0	-		1	1
	M	1 (CNS)	36 Gy; CSI	0		
Case 9	P	1	39.6 Gy; L	1	1	1
	M	0	-			
Case 10	P	0	-		0	0
	M	1 (bone)	21.6 Gy; L	0		
Case 11	P	0			1	0
	M	1 (bone)	36 Gy; L	0		
Case 12	P	1	37.8 Gy; L	1	1	0
	M	0				
Case 13	P	0	-		1	0
	M	1 (bone)	36 Gy; L	1		
Case 14	P	1	36 Gy; L	0	1	0
	M	0	-			
Case 15	P	0	-		O	O
	M	5 (CNS)	40 Gy;CSI	0		
Case 16	P	0			0	0
	M	1	36 Gy; CSI	0		
Case 17	P	1	21.6 Gy; P	0	0	0
	M	0	-			
Case 18	P	1		0	0	0
	M	1 (Bone)	39.6 Gy; L	0		
Case 19	P	1		0	0	0
	M	3 (bone)	36 Gy; L	0		
Case 20	P	0	-		0	0
	M	2 (bone)	36 Gy, L	0		

Gy = Gray; P = irradiation of the primary tumour region; M = irradiation of a metastatic tumour region; L = Local irradiation; CSI = Craino-spinal irradiation; Relapse I Side = Recurrence at an irradiated region; Relapse U.I. Side = Recurrence at a non-irradiated site.

**Table 3:** Overview of higher-grade acute and long-term toxicity in a cohort of 20 patients with relapsed high-risk neuroblastoma.

Acute Toxicity	Long Term Toxicity
hematologic toxicity (N = 10)	myelopathy with paraplegia (N = 2)
Central line infection (N = 1)	secondary outside of the radiation field (N = 1)

**Table 4:** Overview of the biological myelon dose for both 0.1 % and 1.0 % of the myelon volume considering the variable and the constant RBE in both cases with postradiotherapeutic myelopathy.

Case with Myelopathy near T6/7	$D_{0.1\%}\;\left[Gy\left(RBE\right)\right]$	$D_{1.0\%}\left[Gy\left(RBE\right)\right]$
cRBE	35.68	33.56
vRBE	37.65	36.91
Case with myelopathy near T12		
cRBE	22.07	22.03
vRBE	25.20	25.04
Case with myelopathy near T6/7	$D_{0.1\%}\;\left[Gy\left(RBE\right)\right]$	$D_{1.0\%}\left[Gy\left(RBE\right)\right]$
cRBE	35.68	33.56
vRBE	37.65	36.91
Case with myelopathy near T12		
cRBE	22.07	22.03
vRBE	25.20	25.04

## Data Availability

The data presented in this study are available on request from the corresponding author, in compliance with data protection guidelines.
